# *Salmonella *Typhimurium ST213 is associated with two types of IncA/C plasmids carrying multiple resistance determinants

**DOI:** 10.1186/1471-2180-11-9

**Published:** 2011-01-11

**Authors:** Magdalena Wiesner, Edmundo Calva, Marcos Fernández-Mora, Miguel A Cevallos, Freddy Campos, Mussaret B Zaidi, Claudia Silva

**Affiliations:** 1 Departamento de Microbiología Molecular, Instituto de Biotecnología, Universidad Nacional Autónoma de México, Cuernavaca, México; 2 Programa de Genómica Evolutiva, Centro de Ciencias Genómicas, Universidad Nacional Autónoma de México, Apartado Postal 565-A, Cuernavaca, Morelos, México; 3 Hospital General O'Horan y Hospital Regional de Alta Especialidad de la Península de Yucatán, Mérida, Yucatán, México

## Abstract

**Background:**

*Salmonella *Typhimurium ST213 was first detected in the Mexican Typhimurium population in 2001. It is associated with a multi-drug resistance phenotype and a plasmid-borne *bla*_CMY-2 _gene conferring resistance to extended-spectrum cephalosporins. The objective of the current study was to examine the association between the ST213 genotype and *bla*_CMY-2 _plasmids.

**Results:**

The *bla*_CMY-2 _gene was carried by an IncA/C plasmid. ST213 strains lacking the *bla*_CMY-2 _gene carried a different IncA/C plasmid. PCR analysis of seven DNA regions distributed throughout the plasmids showed that these IncA/C plasmids were related, but the presence and absence of DNA stretches produced two divergent types I and II. A class 1 integron (*dfrA12*, *orfF *and *aadA2*) was detected in most of the type I plasmids. Type I contained all the plasmids carrying the *bla*_CMY-2 _gene and a subset of plasmids lacking *bla*_CMY-2_. Type II included all of the remaining *bla*_CMY-2_-negative plasmids. A sequence comparison of the seven DNA regions showed that both types were closely related to IncA/C plasmids found in *Escherichia*, *Salmonella*, *Yersinia*, *Photobacterium*, *Vibrio *and *Aeromonas*. Analysis of our Typhimurium strains showed that the region containing the *bla*_CMY-2 _gene is inserted between *traA *and *traC *as a single copy, like in the *E. coli *plasmid pAR060302. The *floR *allele was identical to that of Newport pSN254, suggesting a mosaic pattern of ancestry with plasmids from other *Salmonella *serovars and *E. coli*. Only one of the tested strains was able to conjugate the IncA/C plasmid at very low frequencies (10^-7 ^to 10^-9^). The lack of conjugation ability of our IncA/C plasmids agrees with the clonal dissemination trend suggested by the chromosomal backgrounds and plasmid pattern associations.

**Conclusions:**

The ecological success of the newly emerging Typhimurium ST213 genotype in Mexico may be related to the carriage of IncA/C plasmids. We conclude that types I and II of IncA/C plasmids originated from a common ancestor and that the insertion and deletion of DNA stretches have shaped their evolutionary histories.

## Background

A substantial amount of the genetic variation in bacteria is carried in plasmids [1]. Plasmids are part of the flexible genome, which is defined by the high plasticity and modularity of its genetic elements and high rates of gene acquisition and loss [2]. They are typically composed of conserved backbone modules coding for replication, maintenance and transfer functions as well as variable accessory modules. The capture of genetic modules by plasmid backbones can increase phenotypic diversity and thereby increase the chances of responding to uncertain environmental changes or of exploiting an opportunity for transient niche expansion [2,3]. Plasmids are classified according to incompatibility (Inc) groups that are based on the inability of plasmids with the same replication or segregation mechanisms to co-exist in the same cell [4]. IncA/C plasmids have attracted the attention of the research community due to their ability to acquire antimicrobial resistance traits and to mobilize them across geographical and taxonomical borders [5]. Recent comparative studies have addressed the evolutionary relationships among the IncA/C plasmids from *Salmonella enterica*, *Escherichia coli*, *Yersinia pestis*, *Yersinia ruckeri*, *Vibrio cholera*, *Photobacterium damselae *and *Aeromonas salmonicida *[5-10]. Over the last decade, increasing attention has been focused on plasmids that harbour the antimicrobial resistance gene *bla*_CMY-2_, which encodes an AmpC-type beta-lactamase that hydrolyzes third-generation cephalosporins [11-13]. In *Salmonella enterica*, *bla*_CMY-2 _is frequently carried by IncA/C or IncI1 plasmids [11,12,14,15].

In a previous study, we examined the genetic variation of a *Salmonella enterica *serovar Typhimurium population isolated from human and food-animal sources from four geographic regions in Mexico [16]. Multilocus sequence typing (MLST) and *Xba*I macro-restriction showed two predominant genotypes, ST19 and ST213. ST19 has been reported worldwide and is the most abundant Typhimurium genotype in the MLST database [17], while ST213 has only been reported in Mexico. Clonal complex analysis supported ST19 as the founder genotype, while ST213 was determined to be a derived genotype replacing ST19. We found a non-random distribution of virulence and antimicrobial resistance accessory genes across chromosomal backgrounds, and several associations among core and accessory genetic markers were detected. First, the *Salmonella *virulence plasmid (pSTV) was found in ST19 strains, but not in ST213 strains. Second, the plasmid-borne *bla*_CMY-2 _gene was found only in ST213 strains. Third, the most abundant integron, the integron profile one (IP-1; *dfrA12*, *orfF *and *aadA2*), was found only in ST213 strains. Fourth, the *Salmonella *genomic island (SGI1) was found in a subgroup of ST19 strains carrying pSTV [16]. The general picture obtained from that study was a population composed of two main genotypes marked by the presence of different accessory genes.

The emergence of the multi-drug resistant (MDR) ST213 genotype associated with resistance to expanded spectrum cephalosporins is a public health threat in Mexico where this clone has rapidly disseminated throughout certain regions, causing severe and fatal infections in infants [18]. The objective of the current study was to examine the association between the recently emerged genotype MDR ST213 and *bla*_CMY-2 _plasmids. ST213 isolates were analyzed by plasmid profiling, PCR replicon typing [19], plasmid *Pst*I restriction profiles [12,20], Southern hybridization, plasmid PCR screening and sequencing of regions scattered throughout the IncA/C plasmids [8], and by their conjugation abilities. We found two divergent types of IncA/C plasmids: one composed of plasmids possessing or lacking the *bla*_CMY-2 _region and the other lacking *bla*_CMY-2_. We discuss our results in the context of epidemiological findings in Mexico, and we present evolutionary hypotheses regarding the origin of the two genetic types of IncA/C plasmids.

## Results

### Plasmid profiling, PCR replicon typing and plasmid size

**Figure 1:**
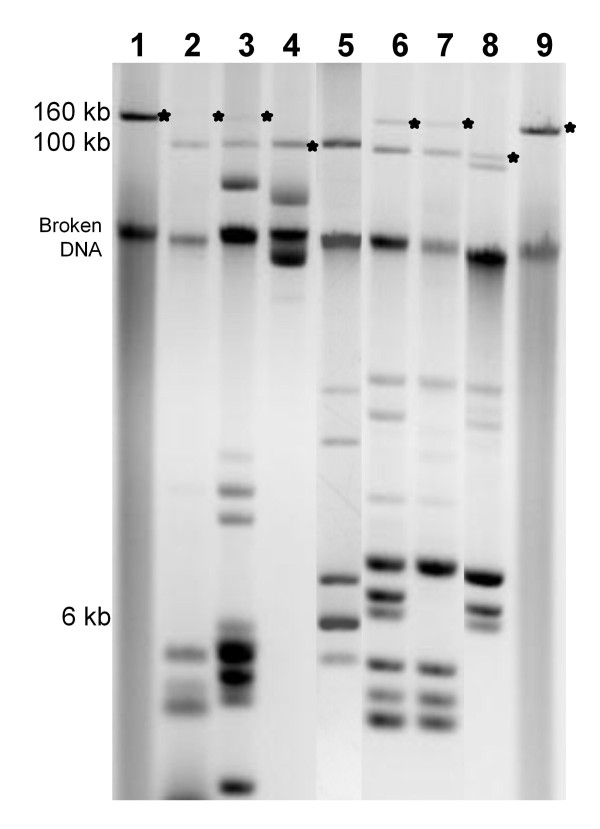
**Electrophoretic plasmid profiles of representative strains of the Typhimurium ST213 genotype**. The diversity of plasmid sizes exhibited by strains carrying or lacking *bla*_CMY-2 _is shown. Lanes 1 and 9 show the *E. coli *reference plasmid pAR060302 [6], which was used as a 160-kb-size marker and as a positive control in the hybridization experiments. Lane 5 shows the plasmid profile of *E. coli *strain E2348/69 used as other size marker (100 and 6 kb) and as a negative control in the hybridization experiments. Lanes 2 to 4 display the plasmid profiles of *bla*_CMY-2_-positive strains belonging to the IncA/C plasmid type I (see Results): YURES 03-7, YUHS 05-78 and YUHS 03-19, respectively. Lanes 6 and 7 show the plasmid profiles of *bla*_CMY-2_-negative strains of plasmid type I: SLRES 02-108 and MIPUS 03-27, respectively, and lane 8 shows the plasmid profile of a representative strain of plasmid type II: SORAPUS 04-29. The IncA/C plasmids are indicated by an asterisk at the right side of the bands.

The plasmid profiles of the 68 ST213 isolates showed that these strains carried a large number of plasmids (one to seven) in a wide range of sizes (3 to 160 kb) (Figure 1).

PCR replicon typing was performed for incompatibility groups that had been reported to be associated with either pSTV or *bla*_CMY-2_, such as IncFII, IncFIB, IncA/C, IncHI2 and IncI1 [14,15,21,22]. All 36 isolates that carried *bla*_CMY-2 _were positive for the IncA/C group and negative for the other Inc groups. Unexpectedly, among the 32 ST213 isolates lacking *bla*_CMY-2_, 23 were positive for the IncA/C group. Additionally, the IncHI2 and IncI1 groups were detected in three and two isolates, respectively. Thirteen *bla*_CMY-2_-negative and IncA/C-positive isolates were selected to represent different sources, states and years of isolation for further analysis, and compared them with the *bla*_CMY-2_-positive isolates (hereafter referred to as CMY- and CMY+, respectively).

**Figure 2:**
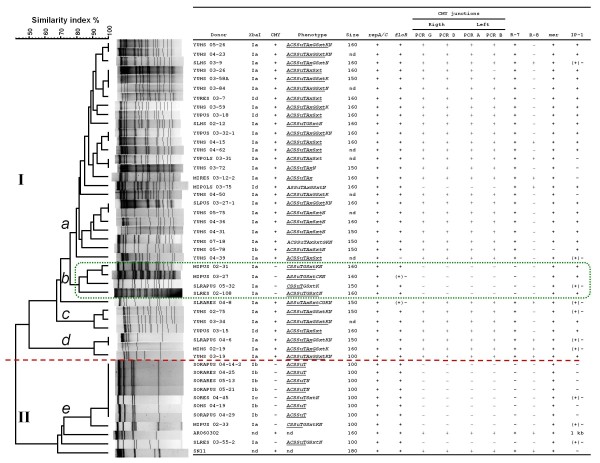
**Dendrogram depicting the genetic relationships between the IncA/C plasmids based on *Pst*I fingerprints**. The dendrogram was constructed with the UPGMA algorithm using Dice coefficients with a 1.0% band position tolerance. The two main groups (designated as types I and II) are separated by a dotted line (similarity index <50%). The five clusters formed at similarity index values >80% are indicated by the letters *a *to *e*. Cluster *b *containing the *bla*_CMY-2_-negative strains within type I is highlighted with a dotted box. The donor column refers to Typhimurium strains used as DNA sources for the transformation of *E. coli *TOP10 or DH5α. The *Xba*I column indicates the cluster name in which the donor strain was placed in the previously published PFGE-*Xba*I restriction dendrogram [16]. The CMY column denotes *bla*_CMY-2_-positive (+) and *bla*_CMY-2_-negative (-) plasmids. The phenotype column describes the resistance phenotype of the donor strain and the resistances transferred by the IncA/C plasmids (underlined). The abbreviations for the antibiotics are described in Methods. The estimated plasmid sizes are indicated in terms of bp. The next ten columns display the results of the PCR screening scheme (Additional file 1, Table S1, Figure 3, Figure 4 and Methods). Positive amplifications are designated by a plus symbol (+) and negative amplifications by a minus symbol (-). In the case of the IP-1 and *floR *columns, the + (-) code indicates that the Typhimurium donor strain was positive for the marker but that the *E. coli *transformants were negative. "1 kb" indicates an integron of around 1,000 bp amplified in pAR060302, as previously described by Call et al. [6]. nd, not determined.

Alkaline lysis profiles and PFGE S1-digestion gels of plasmids from strains in our collection were hybridized with *bla*_CMY-2 _and *repA/C *probes; all of the CMY+ isolates yielded signals in the same plasmids, confirming that *bla*_CMY-2 _is carried in large IncA/C plasmids (100 to 160 kb). In contrast, only the *repA/C *probe hybridized in the CMY- isolates, again targeting large plasmids (100 to 160 kb) (Figure 2). Consistent with their low copy number [9,12,15], the IncA/C plasmids yielded faint bands in the ethidium bromide-stained gels, especially those larger than 100 kb (Figure 1), but they were unambiguously detected in the hybridization experiments.

### Characterization of IncA/C plasmids based on the antibiotic resistance phenotype

To isolate and characterize the IncA/C plasmids present in the Mexican ST213 genotype, *E. coli *TOP10 or DH5α transformants were obtained using plasmid DNA isolated from 32 CMY+ and 13 CMY- strains. Ceftriaxone was used to select CMY+ plasmids, and chloramphenicol was used to select CMY- plasmids because this resistance has been found to be part of the IncA/C plasmid backbone [5,6,8].

All the transformants carrying the IncA/C plasmids also displayed resistance to ampicillin, chloramphenicol, sulphonamides, streptomycin and tetracycline. Resistance to gentamicin was conferred by most of the CMY+ plasmids, and trimethoprim-sulfamethoxazol resistance was mostly detected in the plasmids containing the IP-1 integron (*dfrA12*, *orfF *and *aadA2*; see below). Resistance to neither kanamycin nor nalidixic acid was transferred (Figure 2). These results indicate that the MDR phenotypes of ST213 strains can be explained largely by the presence of IncA/C plasmids.

### *Pst*I restriction fingerprints

The plasmid profiles showed that all of the *E. coli *transformants carried one large plasmid of between 100 and 160 kb. These transformants were analyzed by *Pst*I restriction fingerprinting [12,23]. Cluster analysis of the *Pst*I fingerprints showed two main plasmid types (similarity <50%), which we named type I and type II (Figure 2). All of the CMY+ plasmids were contained in type I and were distributed into three clusters (*a*, *c *and *d*). The CMY- plasmids were found in two distinct groups: one in type II and the other in cluster *b *within type I, suggesting that the CMY- plasmids originated from two divergent IncA/C plasmid types. To put our plasmids into context, IncA/C CMY+ reference plasmids from *E. coli *AR060302 [6] and Newport SN11 [22] were included. The restriction profiles of these plasmids were related to our ST213 type II plasmids, which in contrast were all CMY-.

We compared the sampling information (see Methods) and our previously generated genomic DNA *Xba*I macrorestriction patterns [16] with the plasmid *Pst*I restriction patterns. The observed distribution of the plasmids among genomic backgrounds was consistent with a pattern of clonal spread. The most evident association was between *Xba*I cluster Ib and *Pst*I cluster *e*; these isolates came from Sonora and were sampled in 2004-2005 (Figure 2).

### PCR screening and nucleotide sequence analysis of the plasmids

**Figure 3:**
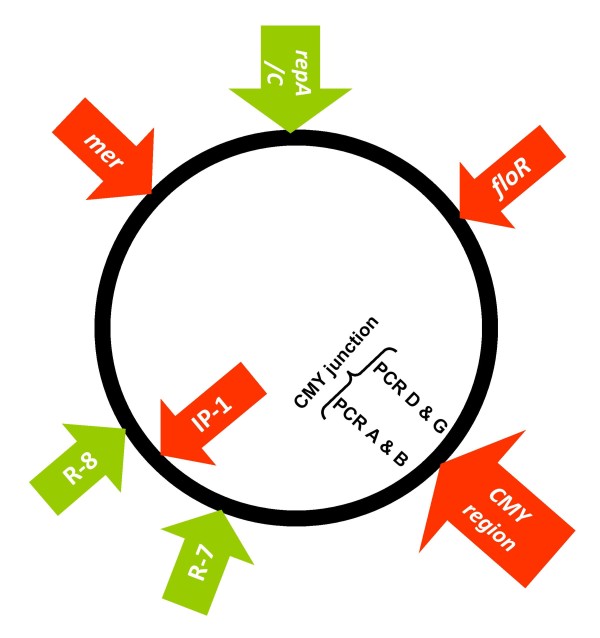
**Schematic representation indicating the relative positions of the molecular markers used to characterize IncA/C plasmids**. Seven regions distributed throughout the reported IncA/C plasmids [5-8,10] were detected by PCR in the 45 ST213 Typhimurium isolates. Antimicrobial resistance determinants are indicated in red. The 2,589-bp *repA/C *region includes the complete *repA *gene, which is involved in plasmid replication and incompatibility group determination. *floR *is a 1,050-bp region spanning almost the complete *floR *gene coding for chloramphenicol resistance. The insertion of the CMY island into the plasmid backbone between *traC *and *traA *was evaluated by PCR D and PCR G for the right junction, and by PCR A and B for the left junction (see Additional file 1, Table S1, Figure 4 and Results). Two regions included in the IncA/C plasmid PCR typing scheme proposed by Welch et al. [8] were analyzed. The 1,431-bp Region 7 (R-7) includes the *bet *gene coding for a phage recombination protein. Region 8 (R-8) is a DNA fragment of 1,600 bp that contains the *dcm *gene coding for a DNA methylase. The presence of the mercury resistance operon (*mer*), frequently associated with the Tn21 transposon [7,8], was evaluated by the amplification of a 2,185-bp region spanning from *merA *to *merT*. The presence of IP-1 (*dfrA12*, *orfF *and *aadA2) *was assessed using primers targeting its conserved sequences.

**Figure 4:**
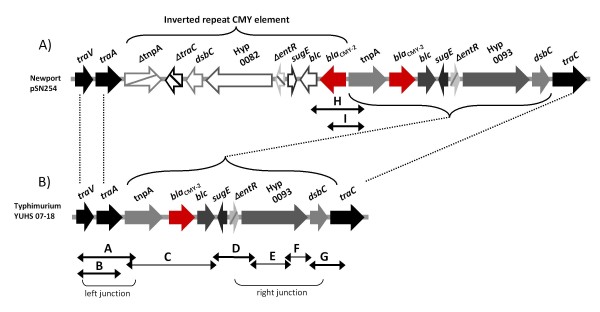
**Schematic diagram of the CMY regions of Newport and Typhimurium**. Panel A shows a schematic diagram of the CMY region of plasmid pSN254 present in Newport [8], which is composed of an inverted repeat CMY element between the *traA *and *traC *genes (unfilled arrows indicate the open reading frames, and the *bla*_CMY-2 _gene is in red). Panel B shows the CMY region of the Typhimurium ST213 strain YUHS 07-18 containing a single CMY element. Truncated genes are indicated by a line crossing the open reading frame arrows. The PCR amplifications designed to map the CMY region are indicated by double arrowheads under the diagrams (see Additional file 1, Table S1 and Results). The PCRs used to screen the CMY junctions are indicated by black double arrowheads.

The *E. coli *transformants were subjected to PCR screening using primer pairs that detect seven regions (*repA/C*, *floR*, CMY region, R-7, R-8, *mer *and IP-1; Figure 3 and Additional file 1, Table S1) distributed throughout the reported IncA/C plasmids [5-8,10]. All the plasmids were positive for the *repA/C*, *floR *and *mer *regions (Figure 2); only one plasmid did not contain the *mer *region (strain YUHS 05-78). The R-7 segment was detected in all the CMY+ plasmids but in none of the CMY- plasmids. We analyzed the CMY region assuming that the right junction would consist of an insertion of *dsbC *upstream of *traC *and that the left junction would consist of an insertion of *tnpA *downstream of *traA *(PCRs G and A, respectively; Figure 4). However, during the nucleotide sequence analysis, we realized that *dsbC *and the hypothetical protein 0093 gene are part of the plasmid core of other closely related IncA/C plasmids lacking the CMY island (see below). Thus, PCR D was also used to detect the insertion of the CMY island at the right junction, demonstrating the insertion of *blc*, *sugE *and Δ*entR *upstream of the 0093 gene (Figure 4). To determine if the flanking region of *traA *is similar in the CMY+ and CMY- plasmids, the left junction was assessed by PCR B (Figure 4). As expected, the CMY- plasmids did not amplify the CMY junctions, whereas most of the CMY+ plasmids amplified the right and left junctions (Figure 2), indicating that with only one exception (strain MIPOLS 03-75), the CMY island is inserted in the same position in these plasmids. The most variable regions of the IncA/C plasmids were the R-8 segment and the IP-1 integron (*dfrA12*, *orfF *and *aadA2*). R-8 was present only in a small fraction of the CMY+ plasmids, including all the plasmids that belong to cluster *d*. Most (25 out of 35) of the *Salmonella *strains that were positive for the IP-1 integron transferred this region along with their IncA/C plasmids. The exceptions were six CMY+ plasmids and four CMY- plasmids (Figure 2). The presence of integrons has been reported for other IncA/C plasmids [6,7,9].

Ten strains representing different geographic locations, years and sources were chosen and their regions analyzed in the PCR screening were sequenced (Additional file 2, Table S2). The sequences were identical for all the plasmids (both CMY+ and CMY-); only the *mer *region showed a single nucleotide substitution (Additional file 2, Table S2). It was surprising that even intergenic regions and third codon positions were invariable. BLAST searches showed that our sequences are identical (100% identity) to the IncA/C plasmids pAR060302 (*E. coli*), peH4H (*E. coli*), pAM04528 (Newport) and pSN254 (Newport); are closely related (99-98%) to the IncA/C plasmids pIP1202 (*Yersinia pestis*), pYR1 (*Yersinia ruckeri*), pP91278 (*Photobacterium damselae*), pP99-018 (*P. damselae*) and pMRV150 (*Vibrio cholerae*); and are related (88-89% identity) to pRA1 (*Aeromonas hydrophila*) [5-10]. The *repA *gene displays the *repA/C*_*2 *_allele described for other IncA/C CMY+ plasmids [19]. Call *et al*. [6] reported that one of the most variable parts of the IncA/C plasmids is the *floR *gene; the *floR *allele found in the ST213 isolates is identical to that of pSN254, which differs from that of pAR060302 by three non-synonymous substitutions. The *traC-dsbC *junction (PCR G) of the CMY island (Figure 4) was found in all the plasmids mentioned above and in the recently described integrating conjugative element ICEPmiJpn1 of *Proteus mirabilis *[GenBank:AB525688]. The finding that *traC-dsbC *is present in pIP1202, pYR1, pP91278, pP99-018, pMRV150 and pRA1, which lack the CMY island, revealed that this gene cluster is part of a conserved core region of these closely related IncA/C plasmids. However, this region did not match with any other plasmids in the database, and it was not amplified in the CMY- plasmids of ST213 (Figure 2). Therefore, to assess the insertion of the right CMY junction, a second marker was used: PCR D spanning from *sugE *to the hypothetical protein 0093 (Figure 4). The complete *traVA-tnpA *right junction (PCR A and B) of the CMY island was identical to that of the *E. coli *and Newport plasmids, but only *traVA *(PCR B) was present in the other CMY- IncA/C reference plasmids. This result indicates that this marker is the left CMY island junction. Interestingly, the ST213 CMY- plasmids did not amplify the *traVA *region, indicating that the region around the CMY island is not present in these plasmids. R-7 and R-8 were found to be present in all the IncA/C reference plasmids, with the only exception being peH4H, which lacks R-7. The *mer *region was detected only in the *E. coli *pAR060302 and Newport plasmids; however, it was found to be related to other *mer *operons present in several plasmids such as pRMH760 (*Klebsiella pneumoniae*).

### Characterization of the CMY region

When we started this study, the only completely sequenced plasmid carrying *bla*_CMY-2 _was that of the Newport strain [GenBank:NC_009140] [8]. PCR mapping experiments were performed to compare the CMY region of our Typhimurium isolates with that of Newport pSN254 (Figure 4 and Additional file 1, Table S1). To determine if the *bla*_CMY-2 _gene is present as an inverted repeat element as in pSN245, we performed PCR H and I, which we expected to produce bands of around 3.2 and 2.3 kb, respectively, based on the *in silico *prediction. The Newport strain SN11 was used as a positive control for these amplifications. No bands were obtained with our Typhimurium isolates, consistent with the notion that our isolates possess only a single *bla*_CMY-2 _gene. We designed a set of primer pairs to amplify overlapping fragments covering the complete CMY region and to obtain the nucleotide sequence of one of our isolates, YUHS 07-18, which is the most recent strain in our collection and which displays *Xba*I and *Pst*I fingerprints prevalent in the ST213 population. The 12,621-bp sequence of YUHS07-18 [GenBank:HQ203988] spanning from *traV *to *traC *was found to be identical to the corresponding region of pSN254 [GenBank:NC_009140] [8], and it has the same CMY region configuration as the *E. coli *plasmid pAR060302 [GenBank:FJ621588] [6].

### Southern blot hybridization of *Pst*I plasmid restriction fingerprints

**Figure 5:**
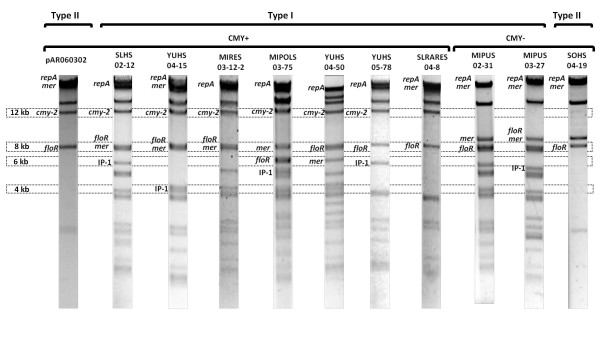
**Representative *Pst*I electrophoretic patterns of ST213 IncA/C plasmids**. The *Pst*I restriction profiles of seven CMY+ strains and three CMY- strains belonging to types I and II are shown. The locations of the genetic markers on the restriction fragments as determined by Southern blot hybridization are indicated. Molecular weight markers are shown at the left side of the figure.

Representative examples of Southern hybridizations of the *Pst*I fingerprints are shown in Figure 5. Hybridization with the *bla*_cmy-2 _probe demonstrated that all CMY+ plasmids were of Giles type A [20], displaying two hybridization bands of about 12 and 0.6 kb. This type has been associated with plasmids that carry one copy of the CMY island, such as pAR060302 [6]. The *repA/C *probe hybridized with the larger band in all the strains, which should be about 55 kb according to an *in silico Pst*I restriction of the complete sequence of pAR060302. This band also hybridized with the *mer *probe for most of the plasmids, in agreement with the *in silico *prediction. However, some polymorphisms were detected using the *mer *probe (Figure 5). The *floR *probe produced a single band of 8 kb, with one exception (Figure 5; MIPOLS 03-75, 7 kb). Finally, hybridizations were performed using the first two genes of IP-1 (*dfr12 *and *orfF*); the *aadA *region was not included in the probe because this gene has been associated with other integrons often present in IncA/C plasmids, such as that of transposon Tn21 [7-9]. Most of the strains produced a hybridization band of 6 kb, but there were polymorphisms (Figure 5).

### Conjugative transfer of IncA/C plasmids

Ten CMY+ and seven CMY- ST213 isolates were evaluated for conjugative transfer of their A/C plasmids to *E. coli *DH5α. Transconjugants were only obtained for the CMY+ strain YUHS 05-78 and at a very low frequency (10^-7 ^to 10^-9^), but they were positive for all nine PCR markers of the donor plasmid, which lacked the *mer *region (Figure 2). However, no transconjugants were observed when an *E. coli *strain carrying the YUHS 05-78 CMY+ plasmid was used as the donor. The highest efficiencies were obtained with a donor:recipient ratio of 1:10 and an incubation for 18 hr on a solid medium (see Methods). In our hands, conjugation efficiencies for AR060302 and SN11 strains were in the order of 10^-5 ^and 10^-6^, respectively. Nevertheless, these frequencies were lower than those reported for these plasmids (i.e. 10^-3^) [6,22].

## Discussion

### Distribution of IncA/C plasmids within Typhimurium genotypes and across geographic regions

We found an association between the Typhimurium ST213 genotype and large IncA/C plasmids. These plasmids accounted for most of the MDR phenotypes of the strains, and they might be related to the ecological success of this recently emerging clone in Mexico. In a previous study, we reported a trend towards the replacement of the founder ST19 by the derived ST213 in four regions of Mexico. We suggested that the carriage of *bla*_CMY-2 _by some of the ST213 strains could only partially explain their increased prevalence because half of the ST213 strains do not harbour *bla*_CMY-2_. In the present study, we discovered that the CMY- strains also harbour large IncA/C plasmids with several resistance determinants. The lack of pSTV and the carriage of IncA/C plasmids are remarkable features of the ST213 genotype in the Mexican Typhimurium population. We speculate that ST213 arose as a clone lacking pSTV and that this condition allowed the acquisition of the large IncA/C plasmids. The success of this association could be due to antimicrobial pressure exerted by human clinical and animal-production practices on the Typhimurium population.

We previously detected several associations among chromosomal genotypes and accessory genes [16], suggesting that the population subgroups generated by these associations could be explained by several evolutionary processes, such as barriers to genetic exchange, genetic drift or recent clonal expansions within the Typhimurium population. The present study reveals the tight association between the ST213 genotype and IncA/C plasmids. Associations between plasmid type and chromosomal genotype have been reported for other *Salmonella *serovars, such as Newport [24,25] and Typhi [26]. Daniels et al. [25] described the relationship between Newport and its plasmids as clonal based on the model proposed by Souza and Eguiarte [3], implying that strong co-evolution may be occurring between the plasmid and the host, with very limited plasmid transfer among bacteria.

The plasmid types appear to cluster geographically. All the Yucatán isolates carry type I plasmids, while all the isolates from Sonora carry only type II plasmids. Isolates from Michoacán and San Luis Potosí harbour plasmids from both types I (CMY+ and CMY-) and II. These patterns demonstrate a distribution gradient of the IncA/C plasmids from the northern (Sonora) to the southern (Yucatán) part of Mexico, with intermediate levels in the middle part of Mexico (Michoacán and San Luis Potosí). This gradient is also related to the higher number of resistances conferred by the type I plasmids than by the CMY- type II plasmids (>6 vs. <6, respectively; Figure 2). These trends provide information for understanding the ecology and epidemiology of the emergent ST213 genotype in Mexico, and they increase our knowledge of the evolution of MDR in Typhimurium.

### Mobility of the ST213 IncA/C plasmids

The conjugation frequencies reported for IncA/C CMY+ plasmids are highly variable. Welch et al. [8] reported a lack of transferability for the Newport IncA/C plasmids, while Poole et al. [22] observed conjugation frequencies between 10^-2 ^and 10^-5^, but only when other replicons were present and co-transferred. On the other hand, most studies have reported that Typhimurium plasmids are non-conjugative [23,27] or transfer at moderate frequencies (10^-3 ^to 10^-7^) [8]. In the present study, we found that only one of the IncA/C plasmids tested was able to conjugate, albeit at very low frequencies (10^-7 ^to10^-9^). The only distinctive feature of this YUHS 05-78, 150 kb CMY+ plasmid is its lack of the *mer *region. It has been suggested that the inability of *Salmonella *CMY+ plasmids to conjugate is due to the insertion of the CMY island into the *tra *operon on the plasmid backbone [22]. However, the conjugative plasmid YUHS 05-78 has the CMY island inserted in between *traA *and *traC*, and this is also true for almost all the other CMY+ plasmids. We think that the reduced conjugative ability of the IncA/C plasmids in *Salmonella *might be due to chromosomally encoded factors, such as the thickness of the cell envelope, rather than to plasmid-encoded features, or it may depend on the presence of additional helper plasmids, as previously suggested [5,8]. The predominant lack of conjugation ability of our IncA/C plasmids agrees with the clonal dissemination trend detected between chromosomal backgrounds and plasmid patterns, as revealed by *Xba*I and *Pst*I digests (Figure 2), respectively. This study provides evidence of frequent rearrangements shaping the genetic composition of the IncA/C plasmids harboured by ST213 strains. It is possible that the IncA/C plasmids circulating in Typhimurium were acquired from other *Salmonella *serotypes or other enteric bacteria such as *E. coli*. The higher plasmid diversity and conjugation frequencies of *E. coli *in comparison with *Salmonella *led Daniels et al. [25] to speculate that insertions and deletions that occur during promiscuous plasmid sharing among *E. coli *isolates occasionally result in plasmids that are successful in *Salmonella*. It is possible that this is the scenario in Mexico, where resistance to ceftriaxone was detected in *E. coli *several years prior to that in *Salmonella *(M. Zaidi, unpublished data).

### Evolutionary origin of the two IncA/C types

The combined results of the PCR screening and the nucleotide sequence analysis suggest that the IncA/C plasmids from types I and II have a recent common origin and are evolving by the insertion/deletion of DNA stretches rather than by point mutations, in agreement with the conclusions derived from other studies [5,6,8,10]. For example, in this study, insertion of the IP-1 integron (*dfrA12*, *orfF *and *aadA2*) and deletion of the R-8 segment were observed in most of the CMY+ plasmids.

The PCR markers and plasmid sizes of the IncA/C CMY+ reference plasmids of *E. coli *AR060302 [6] and Newport SN11 [22] corresponded with those of our Typhimurium IncA/C CMY+ plasmids. However, their *Pst*I restriction profiles were related to type II plasmids, which included most of our Typhimurium IncA/C CMY- plasmids (Figure 2). As the CMY+ reference plasmids and our type II plasmids were found to be similar, we speculate that the ST213 IncA/C CMY+ plasmids diversified from a type II ancestor by the insertion and deletion of DNA segments such as R-8 and the IP-1 integron, among others. Likewise, hybridization assays showed that this integron mapped to some of the bands that are absent in the type II restriction profiles (Figure 5). Despite the nucleotide identity of the sequenced regions of the CMY- plasmids, and aside from IP-1, they share only three of the ten genetic markers (*repA*, *floR *and *mer*; Figure 2 and Figure 3) that have been used to study the IncA/C plasmids, indicating that they belong to an IncA/C plasmid lineage that has not been thoroughly studied yet. The *floR *allele of the CMY+ and CMY- plasmids was identical to that of pSN254, but the CMY region of the CMY+ plasmids was identical to the region of pAR060302, suggesting a mosaic pattern of ancestry with plasmids from other *Salmonella *serovars and *E. coli*. Moreover, the type II CMY- plasmids were found to be smaller (100 vs. 150-160 kb; Figure 2), consistent with the notion that the CMY+ plasmids are the result of the insertion of DNA modules into a type II precursor plasmid. A formal alternative would be that a substantial loss of DNA fragments originally present in the CMY+ plasmids occurred, giving raise to ST213 type II derivatives. In this respect, it would be necessary to obtain the full sequence of some of our CMY+ and CMY- plasmids to identify their genetic compositions and to unravel their evolutionary histories.

## Conclusions

The ecological success of the newly emerging Typhimurium ST213 genotype may be related to the carriage of IncA/C plasmids. Two divergent genetic types of IncA/C plasmids were identified. Type I plasmids are the most abundant and widespread; their genetic compositions are similar to those of other reported IncA/C plasmids. Type II plasmids display a lower number of *Pst*I restriction fragments and are smaller than type I plasmids. Only three of the ten plasmid regions analyzed were detected in type II plasmids, even though the nucleotide sequences for these regions were identical for both types. We conclude that type I and II plasmids originated from a common ancestor and that the insertion and deletion of DNA stretches have shaped their evolutionary histories.

## Methods

### Typhimurium ST213 isolates

The isolates used in the present study were described in a previous publication [16]. Briefly, the isolates were collected from a Mexican surveillance network [28]. The predominant ST213 genotype formed a well-defined group in the dendrogram based on *Xba*I fingerprints (named cluster I), which was subdivided into subclusters Ia, Ib and Ic. The ST213 genotype was associated with the plasmid-borne *bla*_CMY-2 _gene conferring resistance to extended spectrum cephalosporins and with the integron profile one (IP-1) carrying an array of three cassettes containing the genes *dfr12, orfF *and *aadA2 *conferring resistance to trimethoprim and streptomycin. These accessory elements were not present in all the isolates: among the 68 ST213 isolates, 31 lacked *bla*_CMY-2_, 19 did not have IP-1 and 12 lacked both of them. In the present study, a representative sample of 45 isolates was chosen to characterize their IncA/C plasmids. The code labels of the strains were designed to include relevant information about their isolation. The first two letters indicate the state: YU, Yucatán; SL, San Luis Potosí; MI, Michoacán; and SO, Sonora. The third and fourth letters indicate the isolation source: HS, human; PUS, pork meat; RES, beef meat; POLS, chicken meat; RAPUS, pork intestine; and RARES, beef intestine. The first two numbers indicate the year of isolation (from 2002-2007), and the last numbers are the isolate numbers.

### Plasmid DNA extraction and plasmid profiles

Plasmid profiles were obtained by a modified alkaline lysis procedure [29] and were visualized by electrophoresis in 0.7% agarose gels subjected to 60 V for 8 hours. Plasmid profiles of *E. coli *V157 [30], *E. coli *E2348/69 [31] and *E. coli *AR060302 [6] were used as molecular markers for large plasmids, and supercoiled DNA ladders (Invitrogen) were used for smaller plasmids. To resolve plasmids larger than 50 kb, we performed S1 restriction PFGE. Briefly, total DNA was embedded in agarose plugs, and slices were treated with 8 U of nuclease S1 (Promega) at 37°C for 45 min. The PFGE running conditions were 6 V/Cm at 14°C for 15 hours, and switching times ranged from 1 sec to 25 sec. The Low Range PFG Marker was used as the reference standard (New England Biolabs).

### Plasmid transformation and antimicrobial susceptibility testing

Plasmid DNA was introduced into *E. coli *DH5α and TOP10 through electroporation. Transformants were selected on Luria-Bertani (LB) agar containing either 2-μg/ml ceftriaxone for the CMY+ isolates or 15-μg/ml chloramphenicol for the CMY- isolates.

Susceptibility testing was performed by disk diffusion according to Clinical and Laboratory Standards Institute (CLSI) recommendations [32]. The following commercially purchased disks (Becton, Dickinson and Company, Sparks, MD, USA) were used: ampicillin (A), 10 μg; chloramphenicol (C), 30 μg; streptomycin (S), 10 μg; sulfonamides (Su), 250 μg; tetracycline (T), 30 μg; ceftriaxone (Ax), 30 μg; gentamicin (G), 10 μg; trimethoprim-sulfamethoxazole (Sxt), 1.25/23.75 μg; kanamycin (K), 10 μg; nalidixic acid (N), 30 μg. Resistance to ceftriaxone was confirmed by agar dilution using a breakpoint of ≥4 μg/ml.

### Plasmid *Pst*I restriction and Southern hybridization

Plasmid restriction analysis with *Pst*I has been used for the classification of CMY+ plasmids according to Giles types [12,20]. Giles type A has been correlated with IncA/C plasmids carrying a single *bla*_CMY-2 _copy, type B with IncI1 plasmids, and type C with IncA/C plasmids carrying two *bla*_CMY-2 _copies [6,19]. Plasmid DNA was treated with 15 U of *Pst*I (Invitrogen) at 37°C for 6 hours and was electrophoresed in 0.7% agarose for 3 hours at 100 V. The *Pst*I digests were transferred to positively charged membranes (Amersham Hybond™ ^- ^N^+^) and were hybridized with *bla*_CMY-2_, *repA*, *floR*, *merA-T *and *dfr12-orfF *PCR products labeled with α-^32^P-dCTP by standard methods [29]. Hybridizations were carried out at 65°C.

To determine the genetic relationship between the IncA/C plasmids, *Pst*I restriction profiles were analyzed with GelComparII. Clustering was performed using the UPGMA algorithm based on Dice coefficients. One reference isolate was run on all gels. A stringency parameter of 1.0% band position tolerance was used since this was the point at which the common restriction profile was identical across gels.

### PCR assays and nucleotide sequencing

The complete list of primers used in this study is shown in Additional file 1, Table S1. To determine the incompatibility groups of the plasmids, PCR-replicon typing for the *Salmonella *isolates and their *E. coli *transformants was performed using the primers and conditions recommended by Carattoli *et al. *[21]. The incompatibility groups tested were IncA/C, FII, HI1, HI2 and I1.

The *E. coli *transformants carrying the IncA/C plasmids were screened by PCR using primers to detect seven regions distributed throughout the reported IncA/C plasmids [5-8,10] (Figure 3). The primers used are listed in Additional file 1, Table S1, and for a detailed explanation see the legend to Figure 3. The nucleotide sequences of these regions were determined for a representative sample of ten isolates (Additional file 2, Table S2) using the same primers and conditions. Plasmid DNA of the transformants was used for PCR mapping of the CMY island and surrounding regions. Overlapping PCR assays were designed to cover the CMY region using primers previously published [33] or designed by us based on the reported sequence of pSN254 [GenBank:NC_009140] [8]. Nine reactions were designed to determine the configuration at the CMY region (Figure 4, PCRs A-I). PCRs A, B, D and G were included in the plasmid PCR screening scheme to examine the CMY junction of all isolates. The nucleotide sequence for the 12,563 bp CMY region was generated for isolate YUHS 07-18 [GenBank:HQ203988], which was the most recent representative isolate of ST213. Accession numbers of the nucleotide sequences generated for representative strains (Additional file 2, Table S2) are as follows: *repA/C *[GenBank: HQ203980], *floR *[GenBank: HQ203981], PCR G [GenBank: HQ203982], PCR A [GenBank: HQ203983], R-7 [GenBank: HQ203984], R-8 [GenBank: HQ203985], and two *mer *alleles [GenBank: HQ203986] and [GenBank: HQ203987]. All nucleotide sequences were compared against public databases using the BLAST algorithm at NCBI [34].

### Conjugation experiments

We performed conjugation experiments for 17 Typhimurium isolates using a rifampicin (100 μg/ml)-resistant derivative of *E. coli *DH5α as the recipient. We tested several conditions for the conjugation experiments, including a liquid versus solid LB medium, presence of absence of filters, two incubation temperatures (37 and 42°C), several incubation times (from 3 to 48 hrs) and different donor:recipient ratios (1:1, 1:2, 1:5 and 1:10). Conjugations were performed using both the *Salmonella *isolates and their respective *E. coli *transformants. Ceftriaxone (2 μg/ml) and chloramphenicol (15 μg/ml) were used to select for the transfer of CMY+ and CMY- plasmids, respectively. Transfer efficiencies were calculated as the number of transconjugants per donor.

## Authors' contributions

MW and CS conceived the study, performed most of the laboratory work, analyzed and interpreted the data and drafted the manuscript. EC participated in the conception of the study, the interpretation of the data and helped to draft the manuscript. MFM designed the mapping strategy for the CMY region and helped in the laboratory work. MAC participated in the interpretation of data and helped to draft the manuscript. FC performed the antimicrobial susceptibility testing. MBZ provided the strains, helped in the initial conception of the study and in drafting the manuscript. All authors read and approved the final manuscript.

## Supplementary Material

Additional file 1 **Table S1**. Primers used in this study. Click here for file

Additional file 2 **Table S2**. Isolates sequenced and GenBank accession numbers. Click here for file
