# Supplementary material for: Salmonella Typhimurium ST213 is associated with two types of IncA/C plasmids carrying multiple resistance determinants
Source: BMC Microbiol. 2011 Jan 11;11:9. doi: 10.1186/1471-2180-11-9 (PMC3025833; doi:10.1186/1471-2180-11-9)
Supplement: Additional file 1 — Table S1. Primers used in this study. [file 1471-2180-11-9-S1.DOC]

**Table S1. Primers used in this study.**

| **Assay** | **Primer** | **Region** | **Sequence 5’– 3’** | **Size bp** | **Source** |
| --- | --- | --- | --- | --- | --- |
| ***Replicon typing*** | |  |  |  |  |
|  | repA-F | IncA/C | GAG AAC CAA AGA CAA AGA CCT GGA | 300 | [4] |
|  | repA-R |  | TTC TGG AGT TCG TAC AGA GTG AAC |  | [4] |
|  | FII-F | IncFII | ctg tcg taa gct gat ggc | 270 | [2] |
|  | FII-R |  | ctc tgc cac aaa ctt cag c |  | [2] |
|  | HI2-F | IncHI2 | ttt ctc ctg agt cac ctg tta aca c | 644 | [2] |
|  | HI2 -R |  | ggc tca cta ccg ttg tca tcc t |  | [2] |
|  | I1-F | IncI1 | cga aag ccg gac ggc aga a | 139 | [2] |
|  | I1-R |  | tcg tcg ttc cgc caa gtt cgt |  | [2] |
| ***PCR screening*** | |  |  |  |  |
| repA/C | A/C-F | IncA/C | ACT GAA TTC GCG AAA CTG GGG AAA TGT G | 2,589 | This study |
|  | A/C-R |  | TGT GTC GAC GGT TCG TTC GTT GCG TTT CA |  | This study |
|  | A/C-seq |  | CGC AAG AAA GGC GGG AAC GCC AGG TGC |  | This study |
| *floR* | floR-F | *floR* | CCGCGTGGGCCTATACGCTG | 1,100 | This study |
|  | floR-R |  | GAGCCGAAGGAGCACCAGCC |  | This study |
| Region 7 | R7F | R-7 | CAG CAC AAA CAT CTT CCC AGA C | 1,431 | [4] |
|  | R7R |  | GGG TAA CAC CGC CAA CTC TTA C |  | [4] |
| Region 8 | R8F | R-8 | GAA AGC GCA ACA ACA CAA AGA C | 1,600 | [4] |
|  | R8R |  | TGA CTA CTC TTG CCA GCT TTG C |  | [4] |
| *mer* | merA2 | *merAPT* | AAG GTC TGS GCC GCR AGC TTC | 2,185 | [5] |
|  | merA1 |  | ACC ATC GGC GGC ACC TGC G |  | [5] |
|  | merT1 |  | TCT GAT CGC CCT GGG GTT |  | [5] |
|  | merA-F |  | TGATCCCTTCGGCACGGAAG |  | This study |
| integron | CS-F | integron | GGC ATC CAA GCA GCA AG | 2,000 | [1] |
|  | CS-R |  | AAG CAG ACT TGA CCT GA |  | [1] |
|  | orfF-R |  | CTTGCTACGCAAGGGCTAG |  | This study |
| ***CMY region*** | |  |  |  |  |
| PCR A | traV-F | *traV-tnpA* | ATGAAAAATTTGAACATTTTGACCAG | 1,900 | This study |
|  | TnpA2-L |  | TAGCCACTATCCATTCGAAATAAAA |  | [3] |
| PCR B | traV-F | *traV- traA* | ATGAAAAATTTGAACATTTTGACCAG | 1,900 | This study |
|  | traA-R |  |  |  | This study |
| PCR C | TnpA1-L | *tnpA-blc* | ATCAGCTTTTATGACTCGAT | 3,449 | [3] |
|  | Blc-R |  | ATTGGTCTGAAGCTGAGCACT |  | [3] |
| PCR D | sugE-F | *blc*-Hyp | GGGTGAAACAGCCTGGCGCT | 1,497 | This study |
|  | Hyp-R1 |  | AGGTTGGCGGCGTCACATCG |  | This study |
| PCR E | Hyp-F1 | Hyp | CGATGTGACGCCGCCAACCT | 2,331 | This study |
|  | Hyp-R2 |  | CCGTCACCACTGAACAGG |  | This study |
| PCR F | Hyp-F2 | Hyp-*dsbC* | TTCCAAGGTAAAAGTTGGGTCTG | 2,763 | This study |
|  | dsbC-R |  | GGCTTTGAGAGGAATGAACCTTA |  | This study |
| PCR G | dsbC-F | *dsbC-traC* | TAAGGTTCATTCCTCTCAAAGCC | 1,542 | This study |
|  | traC-R |  | GGTGCCGTAATCGAAGATTT |  | [3] |
| PCR H | TnpA2-L | *blc-tnpA* | TAGCCACTATCCATTCGAAATAAAA | 3,255 | [3] |
|  | Blc-R |  | ATTGGTCTGAAGCTGAGCACT |  | [3] |
| PCR I | TnpA2-L | *bla*_CMY-2_-*tnpA* | TAGCCACTATCCATTCGAAATAAAA | 2,298 | [3] |
|  | CMY-R |  | CAG TAG CGA GAC TGC GCA |  | [6] |

**References.**

1. Levesque, C., Piche, L., Larose, C., and Roy, P. H., *PCR mapping of integrons reveals several novel combinations of resistance genes.* Antimicrob. Agents Chemother., 1995. **39**:185-191.

2. Carattoli, A., Bertini, A., Villa, L., Falbo, V., Hopkins, K. L., and Threlfall, E. J., *Identification of plasmids by PCR-based replicon typing.* J. Microbiol. Methods, 2005. **63**:219-228.

3. Kang, M. S., Besser, T. E., and Call, D. R., *Variability in the region downstream of the bla_CMY-2_ beta-lactamase gene in Escherichia coli and Salmonella enterica plasmids.* Antimicrob. Agents. Chemother., 2006. **50**:1590-1593.

4. Welch, T. J., Fricke, W. F., McDermott, P. F., White, D. G., Rosso, M. L., Rasko, D. A., Mammel, M. K., Eppinger, M., Rosovitz, M. J., Wagner, D., Rahalison, L., Leclerc, J. E., Hinshaw, J. M., Lindler, L. E., Cebula, T. A., Carniel, E., and Ravel, J., *Multiple antimicrobial resistance in plague: an emerging public health risk.* PLoS ONE, 2007. **2**:e309.

5. McIntosh, D., Cunningham, M., Ji, B., Fekete, F. A., Parry, E. M., Clark, S. E., Zalinger, Z. B., Gilg, I. C., Danner, G. R., Johnson, K. A., Beattie, M., and Ritchie, R., *Transferable, multiple antibiotic and mercury resistance in Atlantic Canadian isolates of Aeromonas salmonicida subsp. salmonicida is associated with carriage of an IncA/C plasmid similar to the Salmonella enterica plasmid pSN254.* J. Antimicrob. Chemother., 2008. **61**:1221-1228.

6. Wiesner, M., Zaidi, M. B., Calva, E., Fernandez-Mora, M., Calva, J. J., and Silva, C., *Association of virulence plasmid and antibiotic resistance determinants with chromosomal multilocus genotypes in Mexican Salmonella enterica serovar Typhimurium strains.* BMC Microbiol., 2009. **9**:131.
