# Supplementary material for: Salmonella Typhimurium ST213 is associated with two types of IncA/C plasmids carrying multiple resistance determinants
Source: BMC Microbiol. 2011 Jan 11;11:9. doi: 10.1186/1471-2180-11-9 (PMC3025833; doi:10.1186/1471-2180-11-9)
Supplement: Additional file 2 — Table S2. Isolates sequenced and GenBank accession numbers. [file 1471-2180-11-9-S2.DOC]

**Table S2. Isolates sequenced and GenBank accession numbers.**

|  |  |  |  | Region sequenced (bp) | | | | | | | | |
| --- | --- | --- | --- | --- | --- | --- | --- | --- | --- | --- | --- | --- |
|  |  |  |  | *repA/C*  (2,412) | *floR* (1,080) | | PCR A  (1,342) | | PCR G  (1,790) | R-7  (1,331) | R-8  (1,540) | *mer*  (2,179) |
| Isolate | Year, source and State ^a^ | CMY island | *PstI*  cluster | HQ203980 | | HQ203981 | | HQ203983 | HQ203982 | HQ203984 | HQ203985 | HQ203986 |
| YUPUS 03-32-1 | 2003, PM, YU | + | a | + | + | | + | | + | + | - | + |
| YUHS 04-15 | 2004, HE, YU | + | a | + | + | | + | | + | + | + | + |
| YUHS 05-78 | 2005, HS, YU | + | a | + | + | | + | | + | + | - | - |
| YUHS 07-18 | 2007, HE, YU | + | a | + | + | | + | | + | + | - | + |
| MIPOLS 03-75 | 2003, CM, MI | + | a | + | + | | - | | - | + | + | + |
| SLRARES 04-8 | 2004, BI, SL | + | b | + | + | | + | | + | + | + | + |
| MIPUS 03-27 | 2003, PM, MI | - | b | + | + | | - | | - | - | - | + |
| SLRAPUS 04-6 | 2004, SI, SL | + | na | + | + | | + | | + | + | + | + |
| YUHS 03-19 | 2003, HE, YU | + | d | + | + | | + | | + | + | + | + |
| SORES 04-45 | 2004, BM, SO | - | e | + | + | | - | | - | - | - | + |

^a^ YU, Yucatán; SL, San Luis Potosí; MI, Michoacán; SO, Sonora; PM, pork meat; HE, human enteric; HS, human systemic; CM, chicken meat; BI, beef intestine; BM, beef meat.

na, not applicable, this strain was not grouped in a cluster (see Fig. 2).
